# Estrogen and progesterone induce persistent increases in p53-dependent apoptosis and suppress mammary tumors in BALB/c-*Trp53*^+/- ^mice

**DOI:** 10.1186/bcr2094

**Published:** 2008-05-12

**Authors:** Karen A Dunphy, Anneke C Blackburn, Haoheng Yan, Lauren R O'Connell, D Joseph Jerry

**Affiliations:** 1 Department of Veterinary & Animal Sciences and Molecular & Cellular Biology Program, University of Massachusetts, 300 Massachusetts Avenue, Amherst, MA 01003, USA; 2 Current address: John Curtin School of Medical Research, Australian National University, Garran Road, Canberra ACT 0200, Australia; 3 Pioneer Valley Life Sciences Institute, Springfield, 3601 Main Street, MA 01199, USA

## Abstract

**Introduction:**

Treatment with estrogen and progesterone (E+P) mimics the protective effect of parity on mammary tumors in rodents and depends upon the activity of p53. The following experiments tested whether exogenous E+P primes p53 to be more responsive to DNA damage and whether these pathways confer resistance to mammary tumors in a mouse model of Li-Fraumeni syndrome.

**Methods:**

Mice that differ in p53 status (*Trp53*^+/+^, *Trp53*^+/-^, *Trp53*^-/-^) were treated with E+P for 14 days and then were tested for p53-dependent responses to ionizing radiation. Responses were also examined in parous and age-matched virgins. The effects of hormonal exposures on tumor incidence were examined in BALB/c-*Trp53*^+/- ^mammary tissues.

**Results:**

Nuclear accumulation of p53 and apoptotic responses were increased similarly in the mammary epithelium from E+P-treated and parous mice compared with placebo and age-matched virgins. This effect was sustained for at least 7 weeks after E+P treatment and did not depend on the continued presence of ovarian hormones. Hormone stimulation also enhanced apoptotic responses to ionizing radiation in BALB/c-*Trp53*^+/- ^mice but these responses were intermediate compared with *Trp53*^+/+ ^and *Trp*^-/- ^tissues, indicating haploinsufficiency. The appearance of spontaneous mammary tumors was delayed by parity in BALB/c-*Trp53*^+/- ^mice. The majority of tumors lacked estrogen receptor (ER), but ER^+ ^tumors were observed in both nulliparous and parous mice. However, apoptotic responses to ionizing radiation and tumor incidence did not differ among outgrowths of epithelial transplants from E+P-treated donors and nulliparous donors.

**Conclusion:**

Therefore, E+P and parity confer a sustained increase in p53-mediated apoptosis within the mammary epithelium and suppress mammary tumorigenesis, but this effect was not retained in epithelial outgrowths.

## Introduction

Breast cancer is the most frequent type of cancer among women in the US and ranks as the second leading cause of cancer death [1]. Reproductive history predicts the potential for breast cancer development. For instance, early menarche or late menopause increases lifetime exposure to estrogen and both are associated with increased breast cancer risk [2,3]. However, a full-term pregnancy early in reproductive life reduces breast cancer incidence in women by up to 50% [4,5]. Pregnancy also reduces the incidence of carcinogen-induced mammary tumors in rodents [6]. Treatment with estrogen and progesterone (E+P) to mimic serum levels during pregnancy is sufficient to reduce the incidence of carcinogen-induced mammary tumors in rodents [7-9].

Although E+P can render the mammary epithelium resistant to tumors, the underlying mechanisms mediating the protective effect of parity are unknown. It has been proposed that the bolus of ovarian hormones during pregnancy initiates persistent systemic changes in the parous individual. Reductions in growth hormone and prolactin have been associated with decreased breast cancer risk [10-12]. Serum estrogen is also decreased in parous rodents [13]. These changes in the hypothalamic-hypophysial axis would limit the proliferative stimulus to preneoplastic cells and, therefore, reduce the promotional environment for cancer.

Parity also induces changes within the mammary gland, which may prevent tumors. Pregnancy causes morphological changes, including differentiation of the epithelial tree in preparation for lactation, which could alter tumor susceptibility [14]. However, differentiation alone is not sufficient, because prolactin stimulates morphological differentiation but does not render the mammary gland refractory to carcinogen-induced tumors [15]. In addition to morphological differentiation, pregnancy causes permanent changes in the epithelial cells and stroma of the mammary gland [16-18]. Differential expression of growth-regulatory genes such as amphiregulin, pleiotrophin, insulin-like growth factor-1, and transforming growth factor-beta-3 has been reported in parous mammary tissue [19,20] and may limit carcinogenesis in the mammary epithelium.

While alteration in these growth factor signaling pathways may mediate signals from E+P receptors, the p53 pathway appears to be a crucial downstream effector. Both p53 and its downstream transcriptional target, *Cdkn1a *(also known as p21/WAF1), are increased in parous and E+P-treated mammary epithelium in response to 7,12-diamethylbenz [a]anthracene [21]. In the absence of p53, the protection afforded by parity or exogenous E+P is lost [22,23]. Exposure to ionizing radiation causes DNA double-strand breaks and is a risk factor for breast cancer in women [24,25]. Furthermore, p53-dependent responses to ionizing radiation are modest in mammary epithelium of nulliparous mice [26] but are enhanced during pregnancy and by treatment with E+P for 3 days [27]. Breast cancer is the most common tumor among women with Li-Fraumeni syndrome, a syndrome associated with heterozygous mutations in the gene encoding p53 protein [28,29], highlighting the important role of p53 in susceptibility to breast cancer. Whether the susceptibility to breast cancer in Li-Fraumeni syndrome is due to increased risk of loss of heterozygosity for *TP53 *or due to diminished p53 activity is unclear.

In these experiments, we demonstrate that p53-dependent apoptosis is increased by both parity and E+P treatment and that p53 sensitivity is retained after withdrawal of systemic hormones. Hormone-stimulated p53-dependent apoptosis and hormone-induced protection were evident even when p53 activity was reduced because of haploinsufficiency in BALB/c-*Trp53*^+/- ^mice. Consistent with the increased responsiveness of p53, the latency of spontaneous mammary tumors in BALB/c-*Trp53*^+/- ^mice was increased among parous mice compared with nulliparous mice. However, neither enhanced p53 responsiveness nor suppression of tumors was retained in outgrowths from hormone-stimulated BALB/c-*Trp53*^+/- ^mammary epithelium when transplanted into nulliparous hosts.

## Materials and methods

### Animal treatments

BALB/c mice used in these experiments were housed in temperature-controlled facilities with a 12-hour alternating day/night light cycle. All mice were of BALB/cMed genetic background and include *Trp53*^+/+^, *Trp53*^+/-^, and *Trp53*^-/-^. All mice were maintained on AIN76A (Harlan Teklad, Madison, WI, USA) soy-free diet and water *ad libitum*. Six hours prior to sacrifice, animals received either a 5-Gy dose of γ-irradiation from a cesium-137 source (radiation response) or no γ-irradiation (spontaneous response). At sacrifice, the stage of estrus was determined by vaginal smear. The fourth mammary glands were harvested, fixed overnight in 10% neutral-buffered formalin, and embedded in paraffin. All animals were maintained in accordance with procedures approved by the Institutional Animal Care and Use Committee (project #25-09-19). The treatment groups for animals are summarized below.

#### Neonatal E+P or placebo

**Figure 3:**
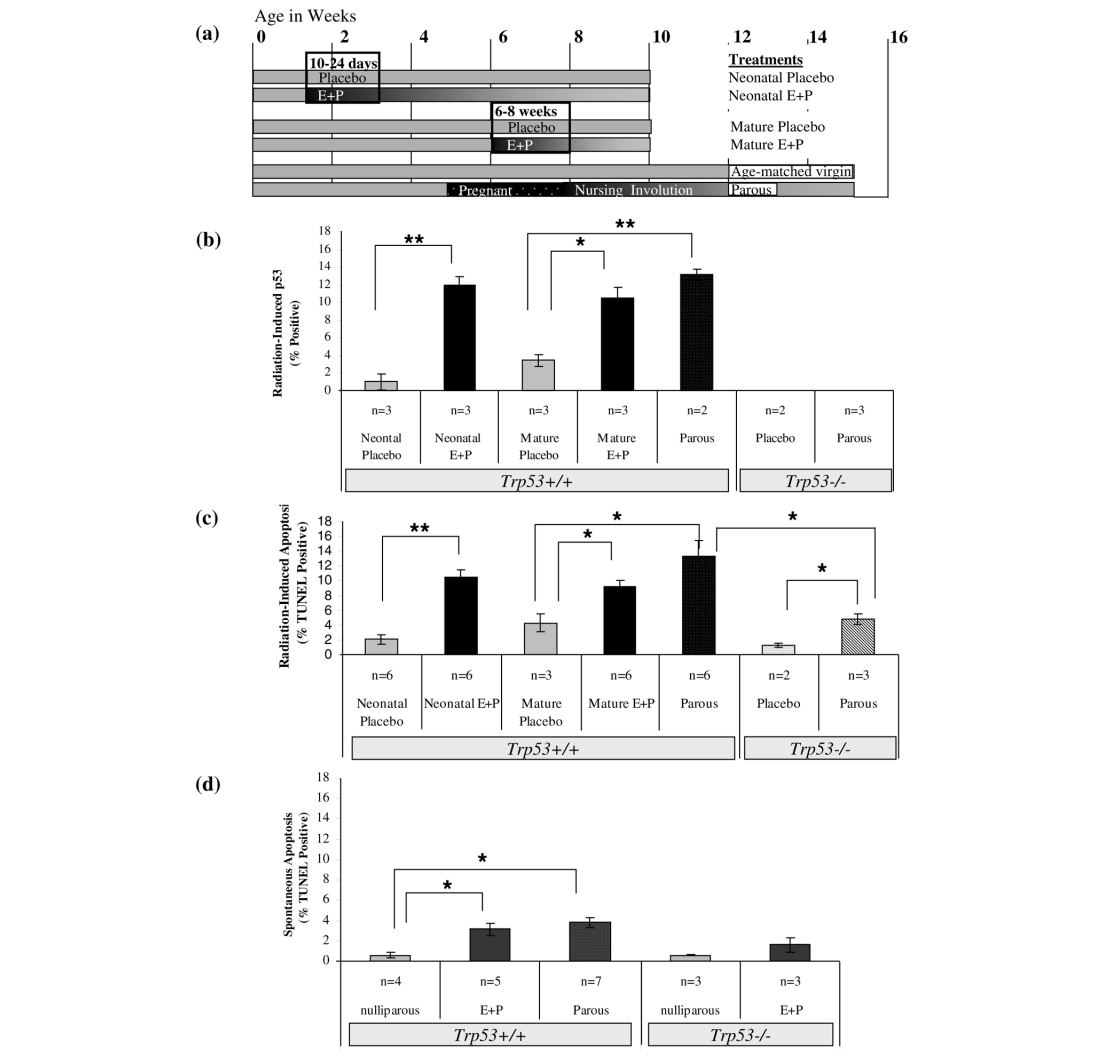
Responses to irradiation in the mammary epithelium are enhanced by hormonal treatments. **(a) **The timeline summarizes treatment schedules of mice. Pellets obtained from Innovative Research of America (Sarasota, FL, USA) were used to deliver either E+P to mice for 14 days (neonatal E+P or mature E+P). Placebo groups were implanted with control pellets at these times. Mice were mated starting at 5 weeks of age and nursed pups for 1 week, and then mammary glands were allowed to involute for 4 weeks post-weaning to produce parous females. Tissues were harvested to determine basal levels of p53 and TUNEL labeling or irradiated 6 hours prior to harvest to determine responses following DNA damage. **(b) **Radiation-induced accumulation of p53 was increased significantly by neonatal E+P and mature E+P compared with the placebo-treated controls. The proportion of p53^+ ^mammary epithelial cells in the E+P-treated groups did not differ from that in parous mice. The tissues from *Trp53*^-/- ^mice were devoid of immunoreactive p53. **(c) **Radiation-induced TUNEL labeling also showed significant increases following treatment with neonatal E+P and mature E+P compared with the placebo-treated controls. Radiation-induced apoptosis was diminished in *Trp53*^-/- ^tissues compared with *Trp53*^+/+^. However, the proportion of TUNEL-positive epithelial cells in parous *Trp53*^-/- ^was increased compared with the placebo-treated *Trp53*^-/- ^controls. **(d) **Levels of spontaneous apoptosis were low in nulliparous mice but increased significantly by neonatal E+P and were similar to the levels observed in tissues from parous mice. No significant difference in TUNEL labeling was detected in *Trp53*^-/- ^tissues from nulliparous and neonatal E+P-treated mice. Bars indicate statistical differences between means: *P < 0.05; **P < 0.01. E+P, estradiol and progesterone; *Trp53*, transformation-related protein 53 (gene in mouse encoding the p53 tumor suppressor protein); TUNEL, terminal uridine deoxynucleotidyl transferase dUTP nick-end labeling.

At 10 days of age, a single 14-day release pellet containing E+P (0.05 mg 17β-estradiol and 3 mg progesterone) or placebo (Innovative Research of America, Sarasota, FL, USA) was implanted subcutaneously as outlined in Figure 3a. Mice were returned to their dams and allowed to mature normally. The fourth mammary glands were harvested at 10 weeks of age.

#### Mature E+P or placebo

At 6 weeks of age, a single 14-day release pellet containing E+P (0.05 mg estradiol and 3 mg progesterone) or placebo (Innovative Research of America) was implanted subcutaneously as outlined in Figure 3a. The fourth mammary glands were harvested at 10 weeks of age.

#### Parous and age-matched virgin

At 5 weeks of age, female mice were mated to produce parous females. After parturition, females were permitted to nurse for 7 days and then were force-weaned and allowed to involute for 28 days. At the time of tissue harvest, parous mice were usually between 14 and 15 weeks of age. Age-matched virgin (AMV) mice controls were also collected between 14 and 15 weeks of age. The timeline for treatment of mice is outlined in Figure 3a.

#### Ovariectomized E+P and nulliparous mice

**Figure 4:**
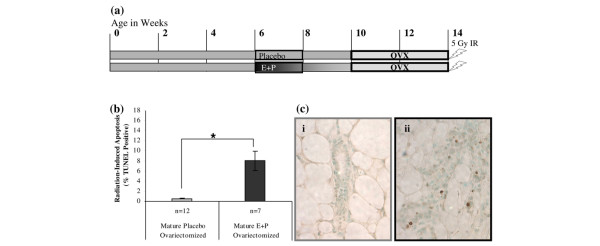
Increases in radiation-induced apoptosis are retained in E+P-treated mice after ovariectomy. **(a) **Mice were treated with E+P or placebo at 6 to 8 weeks of age. At 10 weeks of age, mice were ovariectomized and endogenous hormones were allowed to clear for 2 weeks. All mice received a 5-Gy dose of radiation 6 hours prior to tissue harvest. **(b) **Radiation-induced apoptosis was increased in mammary tissues from E+P-treated mice compared with the placebo-treated controls (*P < 0.01). **(c) **TUNEL-positive epithelial cells were minimal in ducts from nulliparous ovariectomized mice **(i) **but were detected frequently in tissues from mice that were E+P-treated and ovariectomized **(ii)**. All images were taken at × 400. E+P, estradiol and progesterone; IR, ionizing radiation; OVX, ovariectomized; TUNEL, terminal uridine deoxynucleotidyl transferase dUTP nick-end labeling.

BALB/c-*Trp53*^+/+ ^mice were hormone-treated as per mature E+P and placebo-treated mice. At approximately 11 weeks of age, mice were ovariectomized. Endogenous ovarian hormones were allowed to clear for 2 weeks. At approximately 13 weeks of age, all mice received a 5-Gy dose of γ-irradiation 6 hours prior to harvest of the fourth mammary glands. The timeline for the treatments is shown in Figure 4a.

#### Whole organ culture

**Figure 5:**
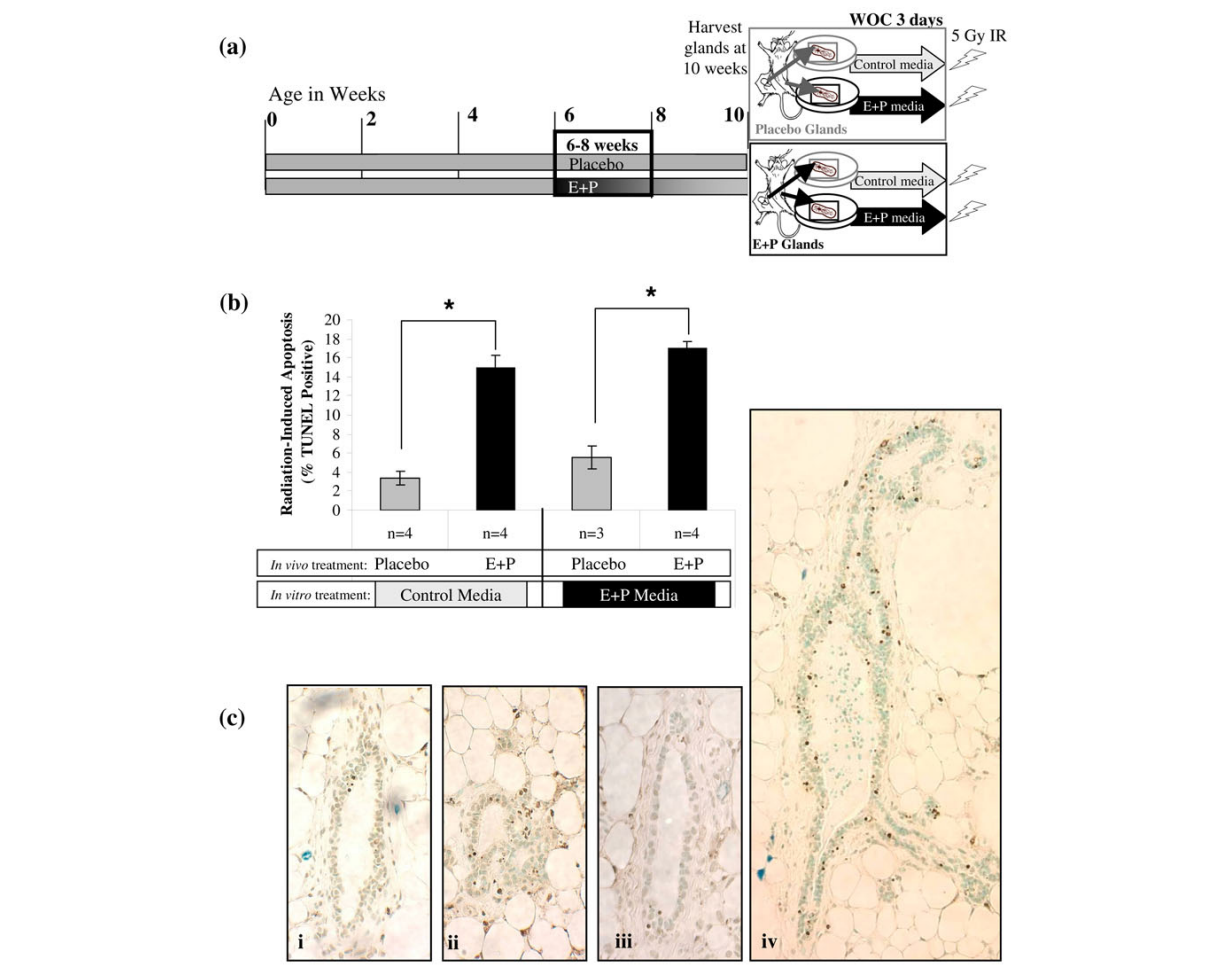
Radiation-induced apoptosis in the mammary epithelium is not dependent on systemic factors. **(a) **Timeline for whole organ culture (WOC) experiments. Mice were treated with placebo or E+P at 6 to 8 weeks of age. At tissue harvest, contralateral fourth mammary glands were placed in either serum-free control media or media supplemented with E+P. Mammary glands were maintained in WOC for 3 days followed by a 5-Gy dose of irradiation 6 hours prior to harvesting and fixing the tissue. **(b) **Mammary glands from mice treated with E+P *in vivo *had a significantly greater apoptotic response to radiation compared with the placebo-treated controls. The increase due to E+P *in vivo *was evident regardless of whether the tissues were maintained in serum-free control media or media supplemented with E+P (*P < 0.01). **(c) **TUNEL-positive staining is shown for placebo-treated glands in control media **(i)**, E+P-treated glands in control media **(ii)**, placebo-treated glands in E+P-treated media **(iii)**, and E+P-treated glands in E+P media **(iv)**. Panels **(i-iii) **were taken at × 400. Panel **(iv) **was taken at × 200 to show that TUNEL-positive cells were present throughout all structures of the E+P-treated glands. E+P, estradiol and progesterone; IR, ionizing radiation; TUNEL, terminal uridine deoxynucleotidyl transferase dUTP nick-end labeling.

BALB/c-*Trp53*^+/+ ^mice were hormone-treated as per mature E+P or placebo. At 12 weeks of age, the fourth inguinal mammary gland was harvested under aseptic conditions, divided into two portions, and floated on Ferrosan surgifoam sponge (Ethicon Inc., Somerville, NJ, USA) in 2 mL of serum-free phenol-red-free Dulbecco's modified Eagle's medium (DMEM)/F-12 (1:1) media supplemented with insulin as described previously [26,27]. The timeline for treatment of mice and tissues is shown in Figure 5a.

#### Spontaneous tumor study

A total of 44 nulliparous and 52 parous BALB/c-*Trp53*^+/- ^mice bred in our facility were monitored for up to 74 weeks for the appearance of tumors. Mammary tumor latency was recorded as the week of first detection by palpation.

#### Epithelial transplant study

**Figure 8:**
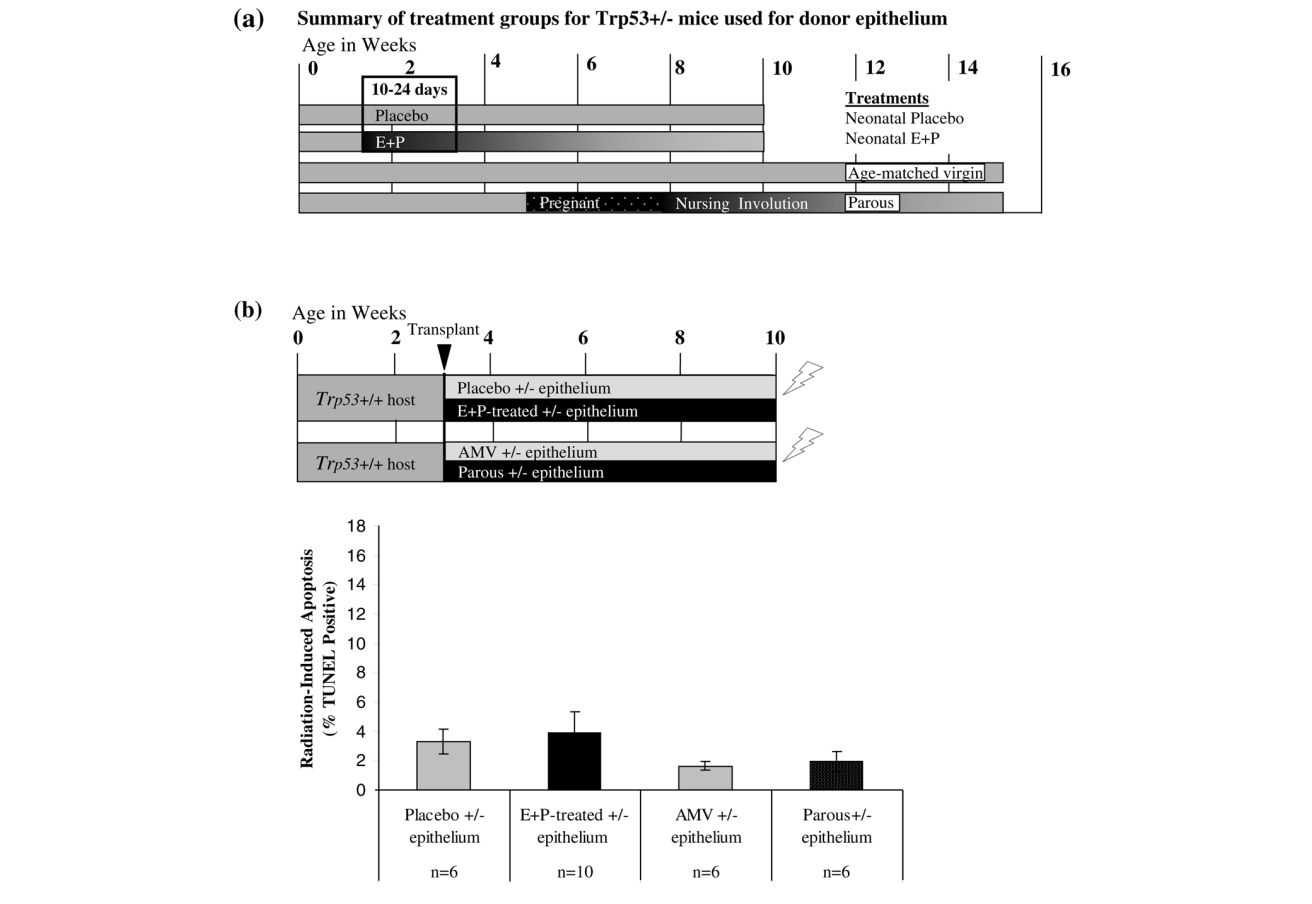
The protective effects of E+P treatment are not retained in epithelial transplant outgrowths. **(a) **Donor tissues were obtained from *Trp53*^+/- ^mice that were treated as summarized in the diagram. One group of mice was implanted with pellets containing either no hormones or E+P. Another group of mice was allowed to undergo a full-term pregnancy or remained unmated to provide age-matched nulliparous tissue. **(b) **Radiation-induced apoptosis was determined in outgrowths of *Trp53*^+/- ^mammary tissue transplanted into wild-type hosts. The timeline of treatments is summarized graphically in the upper panel. The *Trp53*^+/- ^mammary tissues from donors, described in panel (a), were transplanted into mammary fat pads of 3-week-old wild-type hosts and allowed to fill the glands. When 10 weeks old, the transplant-bearing hosts were irradiated 6 hours prior to tissue harvest. Radiation-induced apoptosis was low and did not differ among the mammary epithelial outgrowths from *Trp53*^+/- ^placebo-treated, E+P-treated, age-matched virgin (AMV), or parous mammary tissues. E+P, estradiol and progesterone; *Trp53*, transformation-related protein 53 (gene in mouse encoding the p53 tumor suppressor protein); TUNEL, terminal uridine deoxynucleotidyl transferase dUTP nick-end labeling.

Donor mammary epithelium was obtained from the fourth mammary glands of 12- to 15-week-old BALB/c-*Trp53*^+/- ^female mice that were treated as described above: they were treated neonatally with either E+P or placebo and were parous or AMVs. The timelines for treatment of donor mice are summarized in Figure 8a. The tissues were minced, then placed in cryovials containing 1 mL of DMEM/F-12 supplemented with 10% fetal bovine serum and 10% dimethylsulfoxide, and stored in liquid nitrogen until needed for transplantation.

**Figure 9:**
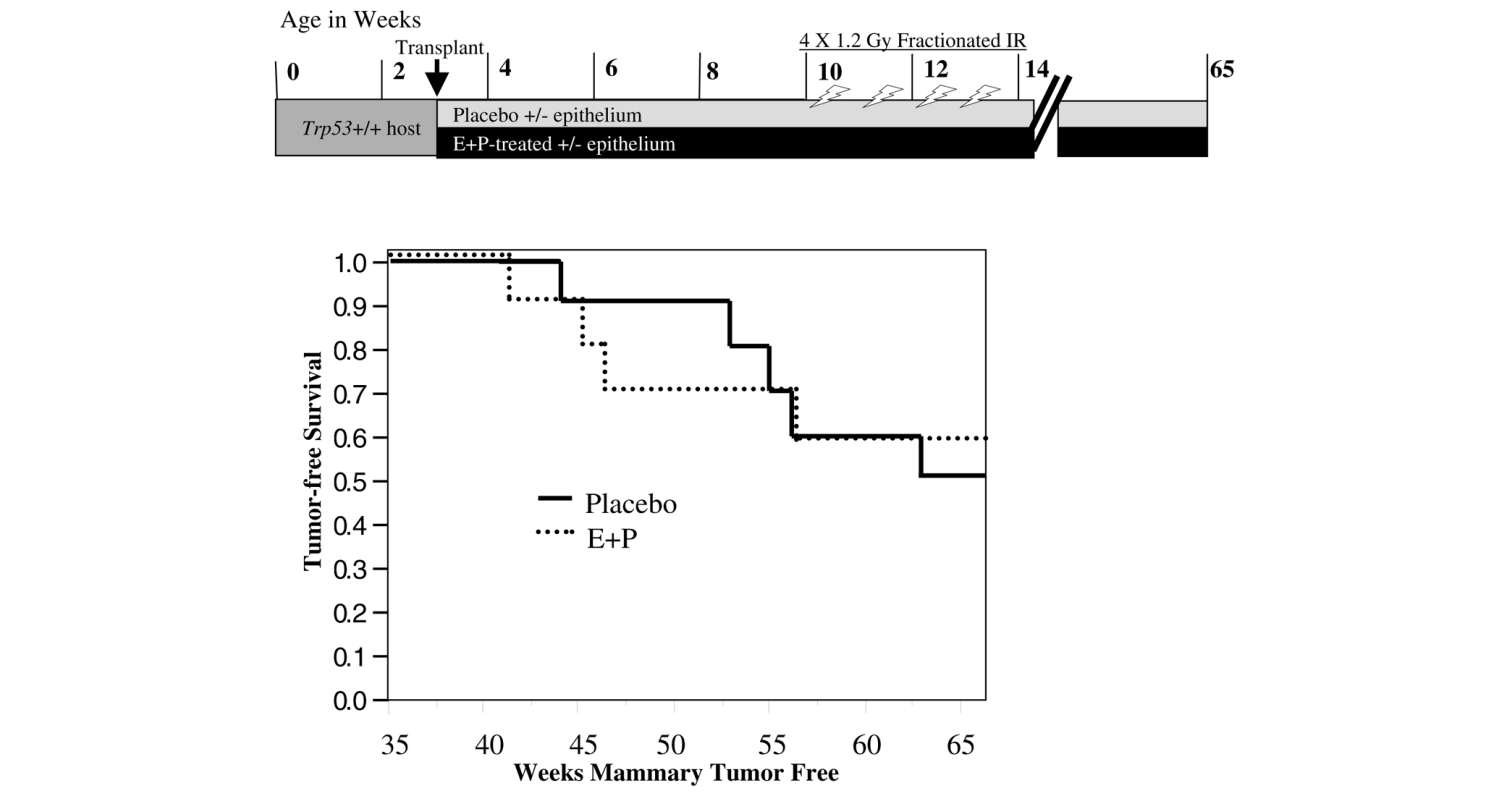
Tumor-free survival in BALB/c mice bearing transplants of *Trp53*^+/- ^mammary epithelium. Tumor incidence was also determined for outgrowths of *Trp53*^+/- ^mammary tissue transplanted into wild-type hosts. The timeline of treatments is summarized graphically in the upper panel. Donor mammary tissues from *Trp53*^+/- ^mice (described in Figure 8a) were transplanted into epithelium-free mammary fat pads of 3-week-old wild-type mice. Beginning at 10 weeks of age, host mice received a 1.2-Gy dose of ionizing radiation once a week for 4 weeks to induce mammary tumors as described previously [30]. Mice were palpated weekly for mammary tumors until 65 weeks old. The incidence and latency of mammary tumors did not differ between epithelial outgrowths from the *Trp53*^+/- ^mammary tissues from E+P-treated donors and nulliparous donors (*P *> 0.05). E+P, estradiol and progesterone; IR, ionizing radiation; *Trp53*, transformation-related protein 53 (gene in mouse encoding the p53 tumor suppressor protein); TUNEL, terminal uridine deoxynucleotidyl transferase dUTP nick-end labeling.

Fragments (~1 mm^3^) of hormone-stimulated epithelium (E+P or parous) and nulliparous epithelium (placebo or AMV) were transplanted into contralateral mammary fat pads cleared of endogenous epithelium of the same host mouse as described previously [22]. The transplant epithelium was allowed to fill the fat pad. At 10 weeks of age, one group of mice bearing transplants was subjected to 5 Gy of γ-irradiation 6 hours prior to tissue harvest for TUNEL (terminal uridine deoxynucleotidyl transferase dUTP nick-end labeling) assay (see timeline in Figure 8b). Another group was subjected to four weekly doses of 1.2 Gy of γ-irradiation [30] and used for the tumor study (see timeline in Figure 9). Animals in the tumor study were monitored by mammary gland palpation for up to 65 weeks. Tumors were removed when less than 1 cm in diameter to allow the continued monitoring of the contralateral gland. At the end of the tumor study, whole mounts were prepared for all remaining mammary glands to assess outgrowths. Transplant efficiency averaged 83%.

### Histological procedures

#### Apoptosis assay

Four-micrometer paraffin-embedded sections were deparaffinized and subjected to TUNEL using a TdT-FragEL DNA Fragmentation Detection Kit (Calbiochem, now part of EMD Biosciences, Inc., San Diego, CA, USA). Tissues were counterstained with methyl green and analyzed for apoptosis by counting 1,200 cells per slide, with the exception of the whole organ culture, where 600 cells per slide were counted.

#### Immunohistochemistry

Immunohistochemistry was performed on a Dako autostainer (Dako, Carpinteria, CA, USA). After antigen-retrieval and blocking steps, 4-μm sections were incubated with primary antibodies recognizing p53 (CM5; Novacastra, now part of Leica Microsystems, Wetzlar, Germany) (diluted 1:200) or estrogen receptor (ER)-α (MC 20; Santa Cruz Biotechnology, Santa Cruz, CA, USA) (diluted 1:200). The signal was amplified using a biotinylated polymer (Vector Laboratories, Burlingame, CA, USA) and detected with diaminobenzidine. Tissues were counterstained with hematoxylin.

#### Histopathology

Tumor tissues were fixed overnight in neutral-buffered formalin, processed, and stained with hematoxylin and eosin for histological assessment. The histological evaluations were conducted using the criteria described for genetically engineered mice [31]. Tissues were considered positive for ERα nuclear staining similar to or exceeding the intensity observed in normal ducts.

#### Whole mount preparation

Whole mammary glands were fixed in Carnoy's fixative (6:3:1 ethanol/chloroform/glacial acetic acid) and then stained in carmine alum solution (2 g/L carmine; 10 mM aluminum potassium). Glands were dehydrated in graded ethanol, cleared in xylenes, and mounted on slides with permount.

#### Microscopy

A Spot digital camera (Diagnostic Instruments, Sterling Heights, MI, USA) mounted on a Zeiss Axioscope microscope (Carl Zeiss, Jena, Germany) was used to acquire figures. The magnification is noted for each image.

### Statistical analysis

#### Quantification of TUNEL and p53

Thresholds for detection were set to determine positive staining for p53 and TUNEL using BIOQUANT Imaging software (BIOQUANT Image Analysis Corporation, Nashville, TN, USA). A total of 1,200 mammary epithelial cells were counted for each specimen, except for whole organ cultures, where 600 cells per specimen were counted. The two-sided Student *t *test was used to determine differences between groups. A *P *value of less than 0.05 was considered significantly different. Error bars indicate the standard error of the mean.

Analysis of variance was used to test the effects of *Trp53 *genotype and hormone treatments on apoptosis. Survival of *Trp53*-deficient mice was limited in some treatments, due to spontaneous nonmammary tumors that are common in these mice. Nonparametric Kruskal-Wallis and normal theory analysis of variance were performed to determine the reasonableness of pooling data within control groups (AMV, neonatal placebo, mature placebo) and within the hormone-stimulated groups (parous, neonatal E+P, mature E+P). Both the one-way Kruskal-Wallis nonparametric test of medians and the one-way analysis of variance test of means were used to analyze the main effects of *Trp53 *genotype and hormone treatment as well as the interactions. The Wilcoxon rank sum test was used to examine the significance of differences between means.

#### Survival curves

The tumor-free survival data were analyzed using survival distribution with censoring in JMP IN (Duxbury Press, Pacific Grove, CA, USA). The differences in tumor incidences were determined by the chi-square test, and differences were considered statistically significant for *P *values of less than 0.05.

## Results

### E+P treatment mimics the effects of parity on responses to ionizing radiation

**Figure 1:**
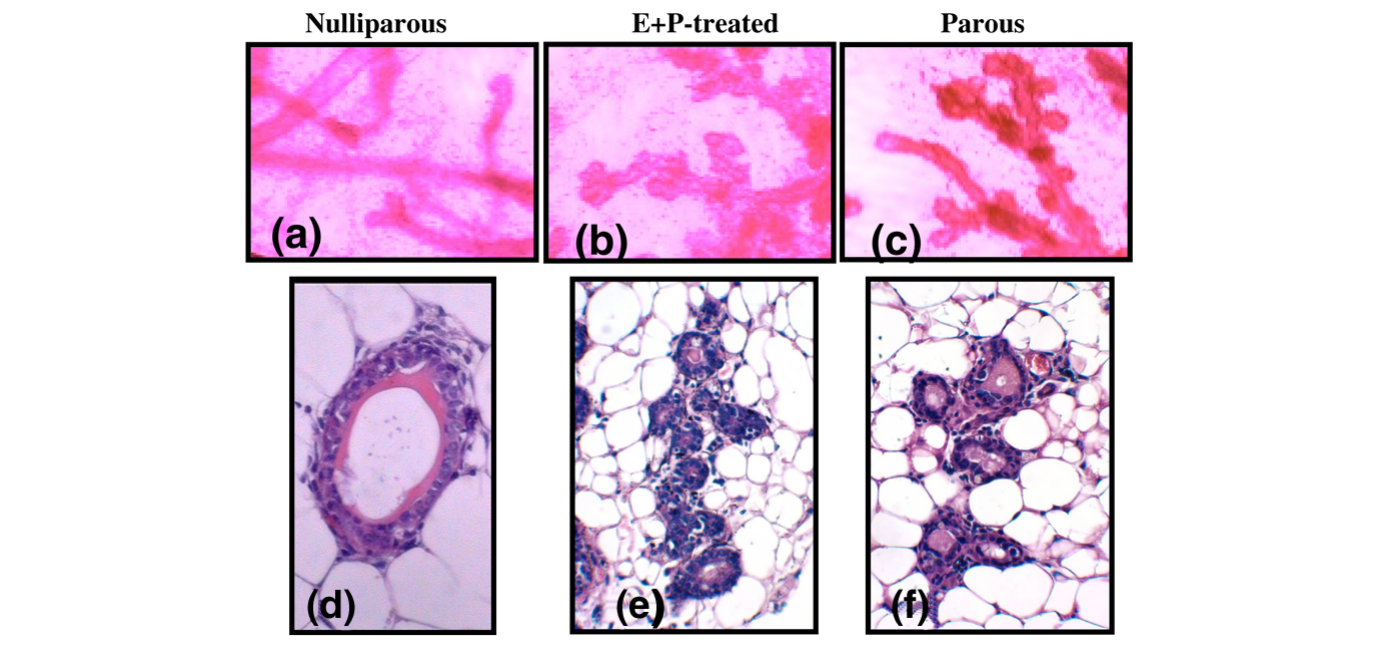
Changes in mammary gland architecture induced by estrogen and progesterone. Tissues from mice that were nulliparous **(a, d)**, E+P-treated **(b, e)**, and parous **(c, f) **were examined using whole mounts **(a-c) **and 4-μm sections stained with hematoxylin and eosin **(d-f)**. Magnifications, × 4 **(a-c) **and × 100 **(d-f)**. E+P, estrogen and progesterone.

Treatment with E+P for mature (6 to 8 weeks of age) or neonatal (10 to 24 days of age) mice resulted in lobular-alveolar development along the ductal tree and histological features that were indistinguishable from the glands from parous-involuted mice (Figures 1b and 1e versus 1c and 1f). However, the architecture of mammary glands from E+P-treated and parous mice differs from that of glands of 10-week-old nulliparous mice (Figures 1a and 1d). Therefore, treatment with E+P mimics the effects of parity on mammary gland architecture.

**Figure 2:**
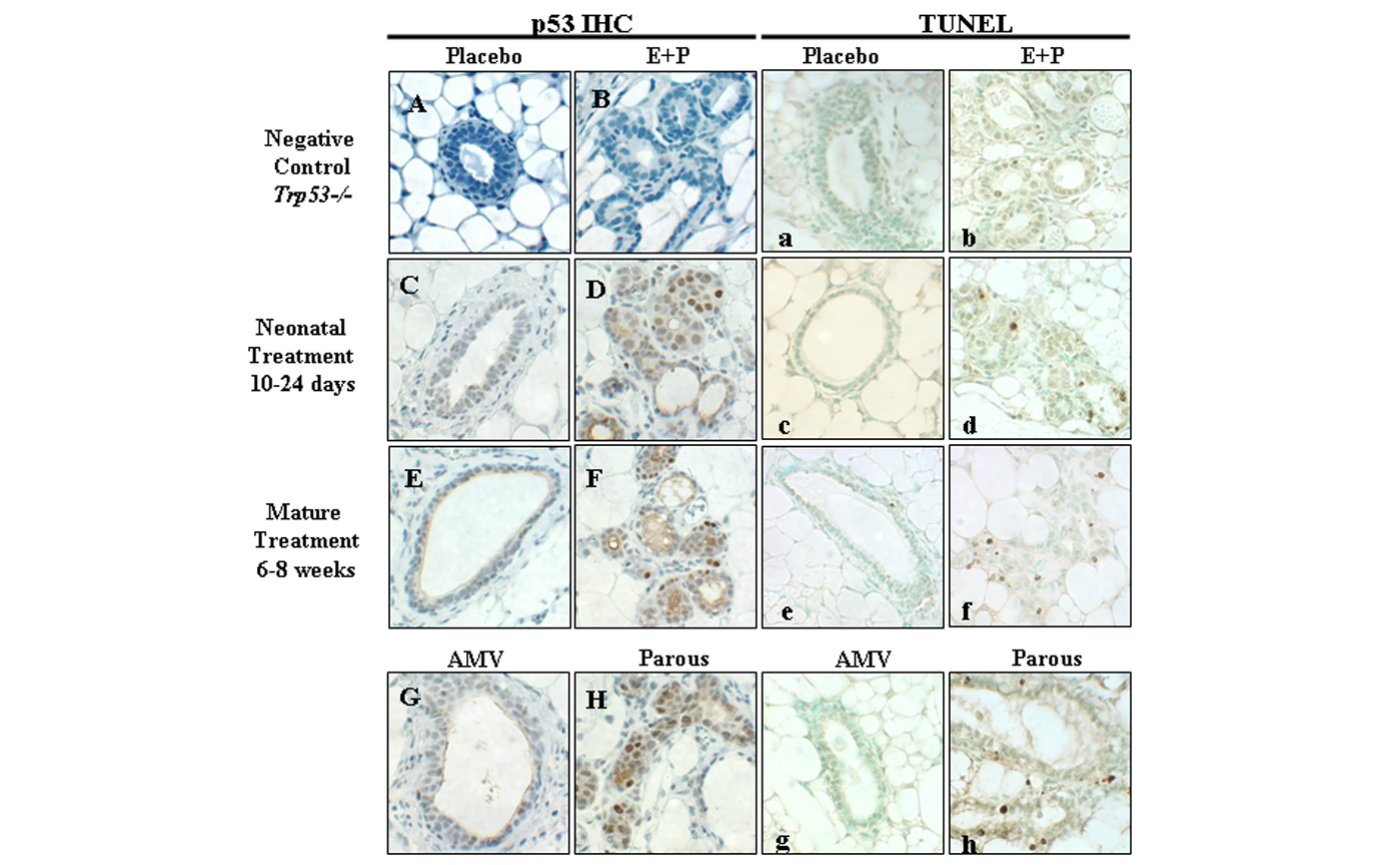
Radiation-induced accumulation of p53 protein **(A-H) **and apoptosis **(a-h) **in mouse mammary glands. *Trp53*^+/+ ^mice were treated with placebo or E+P either neonatally (10 to 24 days old) or when mature (6 to 8 weeks) and then were irradiated to examine nuclear accumulation of p53 and TUNEL labeling as indicators of p53 activity. Responses were also examined in mammary tissues from nulliparous mice (AMV = age-matched virgin with parous) and following a full-term pregnancy (parous). The absence of staining in *Trp53*^-/- ^tissues ensures the specificity of p53 immunostaining **(A, B) **and p53 dependence of TUNEL staining **(a, b) **in placebo- and E+P-treated mice, respectively. Mammary glands from *Trp53*^+/+ ^mice treated with placebo, either neonatally or when mature, exhibited low levels of p53 and TUNEL labeling – **(C**, **E) **and **(c**, **e)**, respectively – and were similar to age-matched virgins **(G, g)**. In contrast, both p53^+ ^nuclei and TUNEL labeling were prevalent in the mammary tissues from *Trp53*^+/+ ^mice treated with E+P either neonatally or at maturity – **(D**, **d) **and **(F**, **f)**, respectively – and were similar to glands from parous mice **(H, h)**. All images were taken at × 400. E+P, estradiol and progesterone; IHC, immunohistochemistry; *Trp53*, transformation-related protein 53 (gene in mouse encoding the p53 tumor suppressor protein); TUNEL, terminal uridine deoxynucleotidyl transferase dUTP nick-end labeling.

Prior to ionizing radiation, expression of p53 protein was low in the mammary epithelium. Six hours after ionizing radiation, weak nuclear accumulation of p53 protein was observed in a majority of mammary epithelial cells in tissues from placebo-treated mice (Figures 2C and 2E). In contrast, strong nuclear p53 staining was observed in mammary tissues from mice that had received E+P either during the neonatal period or when mature (Figures 2D and 2F, respectively). Similarly, tissues from age-matched nulliparous mice had lower levels of p53 staining compared with parous mice (Figures 2G and 2H, respectively). The staining is specific as *Trp53*^-/- ^tissues lacked detectable p53 (Figures 2A and 2B).

For quantification, thresholds were set so that the subset of cells with strong nuclear accumulation were scored as positive. The percentage of cells with p53 accumulation was consistently greater in mammary epithelium following hormonal exposures (Figure 3b). Nuclear accumulation of p53 was more frequent among mice treated neonatally with E+P compared with placebo (*P *< 0.01). Likewise, p53 expression in the mammary epithelium of mature E+P-treated mice and parous mice was 3- to 4-fold greater than that of mature placebo-treated mice (*P *< 0.05). There is no statistical difference in expression of p53 protein in the mammary epithelium between neonatally E+P-treated, mature E+P-treated, or parous mice. Likewise, there is no statistical difference in expression of p53 protein between the two placebo groups.

Apoptosis in the mammary epithelium of placebo-treated, E+P-treated, and parous mice provides a surrogate measure of p53 activity. As for p53, radiation-induced TUNEL-positive nuclei were infrequent in the mammary epithelium of placebo-treated and age-matched virgin mice (Figures 2c, 2e, and 2g) compared with mice treated with E+P or mice that were parous (Figures 2d, 2f, and 2h). The lobular and ductal epithelium appear to be similarly sensitized to apoptosis because TUNEL-positive cells were distributed similarly throughout the gland. These responses were p53-dependent as radiation-induced apoptosis was reduced substantially in *Trp53*^-/- ^mice (Figures 2a and 2b).

Apoptotic responses to radiation were increased to similar extents by E+P regardless of whether it was administered neonatally or to mature mice and did not differ from radiation-induced apoptosis detected in parous mice (Figure 3c). The percentage of apoptosis did not differ between the placebo-treated mice and AMV mice, so aging is not a factor in acquiring enhanced apoptotic response to radiation (data not shown). Radiation-induced apoptotic responses in the mammary epithelium from nulliparous *Trp53*^-/- ^mice were low and not significantly different from the apoptotic response in the *Trp53*^+/+ ^neonatal placebo or mature placebo, suggesting that p53 response is largely absent in nulliparous mammary tissue (Figure 3c). However, in *Trp53*^-/- ^parous mammary epithelium, apoptotic response to radiation was significantly greater than in *Trp53*^-/- ^nulliparous mice, indicating that parity can enhance apoptosis via p53-independent mechanisms. Though elevated, the responses in *Trp53*^-/- ^parous glands are 2.5-fold lower than *Trp53*^+/+ ^parous glands (4.0% versus 11.0%, respectively; *P *< 0.05), indicating that a majority of the response in tissues from *Trp53*^+/+ ^mice is p53-dependent. Spontaneous apoptosis was also compared for the *Trp53*^+/+ ^and *Trp53*^-/- ^genotypes. Basal levels of apoptosis in *Trp53*^+/+ ^tissues were significantly increased in E+P-treated and parous mice compared with nulliparous mice (Figure 3d). Basal levels of apoptosis were also detected in *Trp53*^-/- ^tissues, but there was no statistical difference between nulliparous and E+P-treated mice.

Both the results for radiation-induced p53 accumulation and TUNEL-positive epithelial cells demonstrate that exposure to E+P results in an increase in p53 responsiveness to radiation that is similar to the effect of parity and that is sustained for at least 7 weeks beyond hormone exposure and that E+P treatment is effective regardless of the age at which mice are treated. The majority of the apoptosis in hormone-stimulated tissues depends on p53, but some p53-independent radiation-induced apoptosis was also detected.

### Enhanced apoptotic responses are retained following withdrawal of estrogen and progesterone

To test whether endogenous E+P is necessary to maintain the elevated apoptotic responses to radiation in E+P-treated epithelium, nulliparous females were treated with E+P or placebo pellets at 6 to 8 weeks of age (Figure 4a). The mammary tissues were allowed to regress for 2 weeks following the hormone treatments and then the mice were ovariectomized. The mice were allowed to recover for 2 weeks following ovariectomy to clear endogenous hormones. Radiation-induced apoptosis was retained in the E+P-treated mice even after ovariectomy (Figures 4b and 4c). The radiation responses did not differ from ovary-intact animals. These results imply that E+P treatment induces chronic changes in p53-dependent apoptotic responses to ionizing radiation and continued exposure to ovarian hormones is not necessary.

To determine whether effects of hormone stimulation were intrinsic to the mammary gland, whole mammary glands from placebo- and E+P-treated mice were cultured in control media or media supplemented with E+P (Figure 5a). Radiation-induced apoptosis in the nulliparous mammary epithelium was low in control media and increased modestly in E+P media (Figures 5b and 5c, i and iii). In contrast, mammary glands from mice that had been treated with E+P *in vivo *had statistically greater apoptotic response to irradiation in both control and E+P media (Figures 5b and 5c, ii and iv). Thus, increased responsiveness to radiation is intrinsic to hormone-treated mammary tissues and is retained even after removal from systemic influences by placing tissues in whole organ culture.

Taken together, these data demonstrate that E+P-treated mammary glands are primed to be more responsive to irradiation than the nulliparous glands and that this response is independent of the continued presence of ovarian hormones (Figure 4b) or systemic factors (Figure 5b). The high apoptotic response of the E+P-treated glands occurs equally in main branching ducts (Figure 5c, iv) as it does in the lobulo-alveolar structures. Thus, the apoptotic response to irradiation in all of these experiments is not specific to a particular structure within the mature mammary gland.

### Parity is protective in the BALB/c-*Trp53*^+/- ^mice

**Figure 6:**
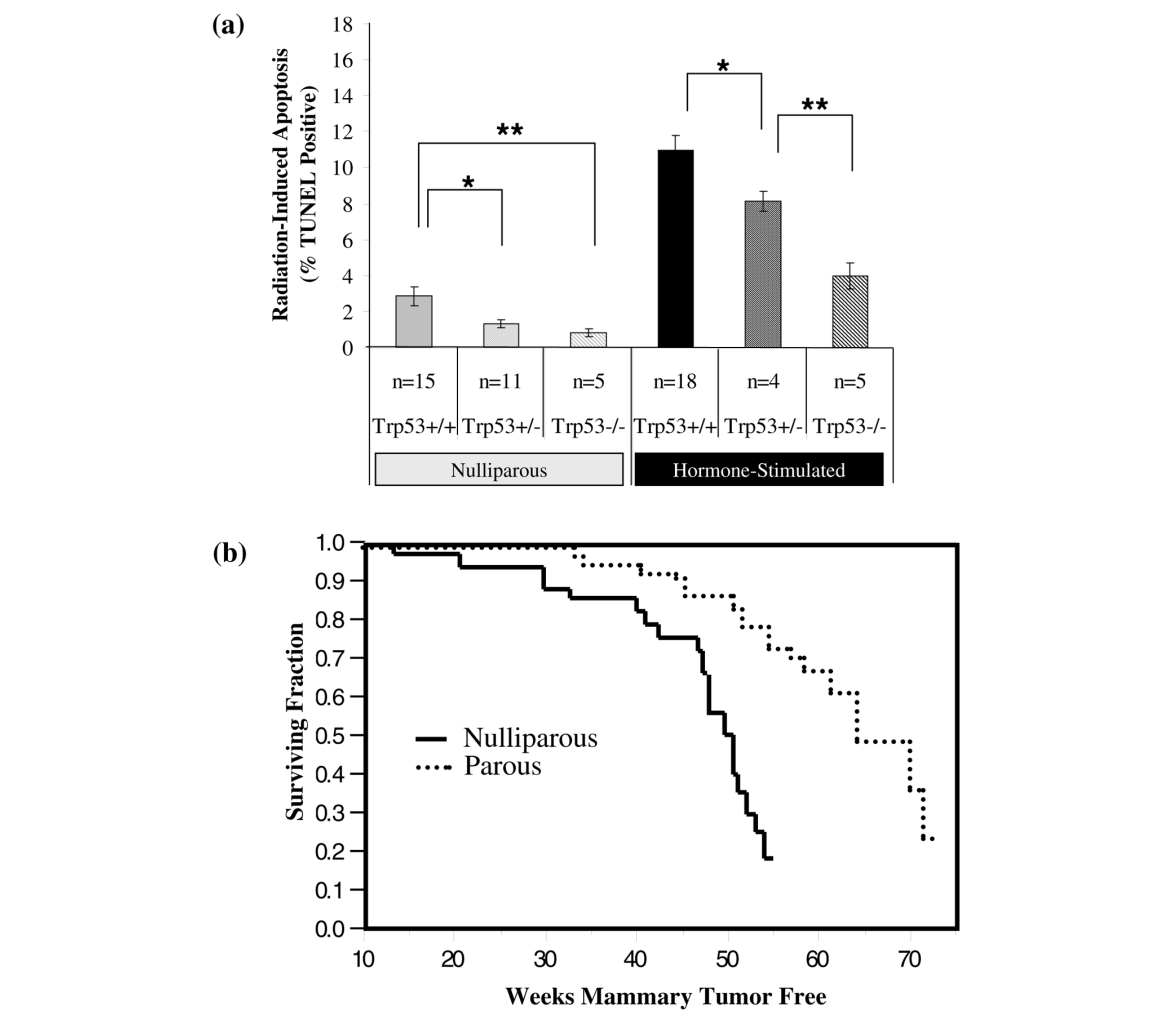
Effects of hormonal treatments on apoptosis in BALB/c-*Trp53*^+/- ^mammary glands. **(a) **Levels of TUNEL-positive mammary epithelium from nulliparous mice were decreased significantly with dosage of *Trp53 *(*Trp53*^+/+ ^> *Trp53*^+/- ^> *Trp53*^-/-^) in both nulliparous and hormone-stimulated groups. Data from control groups and hormone-treated groups of mice were pooled to provide sufficient numbers for all genotypes. The nulliparous group represents placebo-treated and age-matched virgins. The hormone-stimulated group represents mammary tissues from mice treated with E+P and parous mice as described in Figure 2a. Bars indicate statistical differences between means: *P < 0.05; **P < 0.01.**(b) **The incidence of mammary tumors was monitored in both nulliparous and multiparous BALB/c-*Trp53*^+/- ^female mice. The latency of spontaneous mammary tumors was increased in parous compared with nulliparous mice (*P *< 0.001). TUNEL, terminal uridine deoxynucleotidyl transferase dUTP nick-end labeling.

BALB/c-*Trp53*^+/- ^mice develop spontaneous mammary tumors, providing a model for breast cancer in Li-Fraumeni syndrome [32]. Therefore, we compared the effects of *Trp53 *genotype and hormonal treatments on radiation-induced apoptosis in mammary tissues. As no differences in responses were detected within the nulliparous control groups (AMV, neonatal placebo, mature placebo) or within the hormone-stimulated groups (parous, neonatal E+P, mature E+P), the data for these groups were pooled (as described in Materials and methods). This was necessary due to the spontaneous nonmammary tumors that are common among the *Trp53*-deficient mice and limited survival. The overall effect of *Trp53 *genotype was significant in both the nulliparous control and hormone-stimulated groups (Kruskal-Wallis test, *P *= 0.04 and 0.002, respectively). In the nulliparous group, responses in the *Trp53*^+/+ ^mice were significantly greater than either *Trp53*^+/- ^or *Trp53*^-/- ^(Figure 6a). Apoptotic responses were also decreased significantly with decreasing dosage of *Trp53 *in the hormone-stimulated mice (*P *< 0.05). Thus, *Trp53*^+/- ^mammary tissue is haploinsufficient with respect to radiation-induced apoptosis, but p53 activity can be increased significantly in *Trp53*^+/- ^mammary epithelium following hormonal stimulation.

To test whether the increased activity of p53 following hormonal stimulation alters tumorigenesis, we compared incidence and latency of spontaneous mammary tumors between nulliparous *Trp53*^+/- ^mice and parous *Trp53*^+/- ^mice. Mammary tumors occurred in 43.2% of the nulliparous mice and 34.6% of the parous mice. Mammary tumor-free survival time was increased by 15 weeks in the parous group (Figure 6b) (*P *< 0.0001). Therefore, parity is effective in protecting from mammary tumors in this mouse model of Li-Fraumeni syndrome.

**Figure 7:**
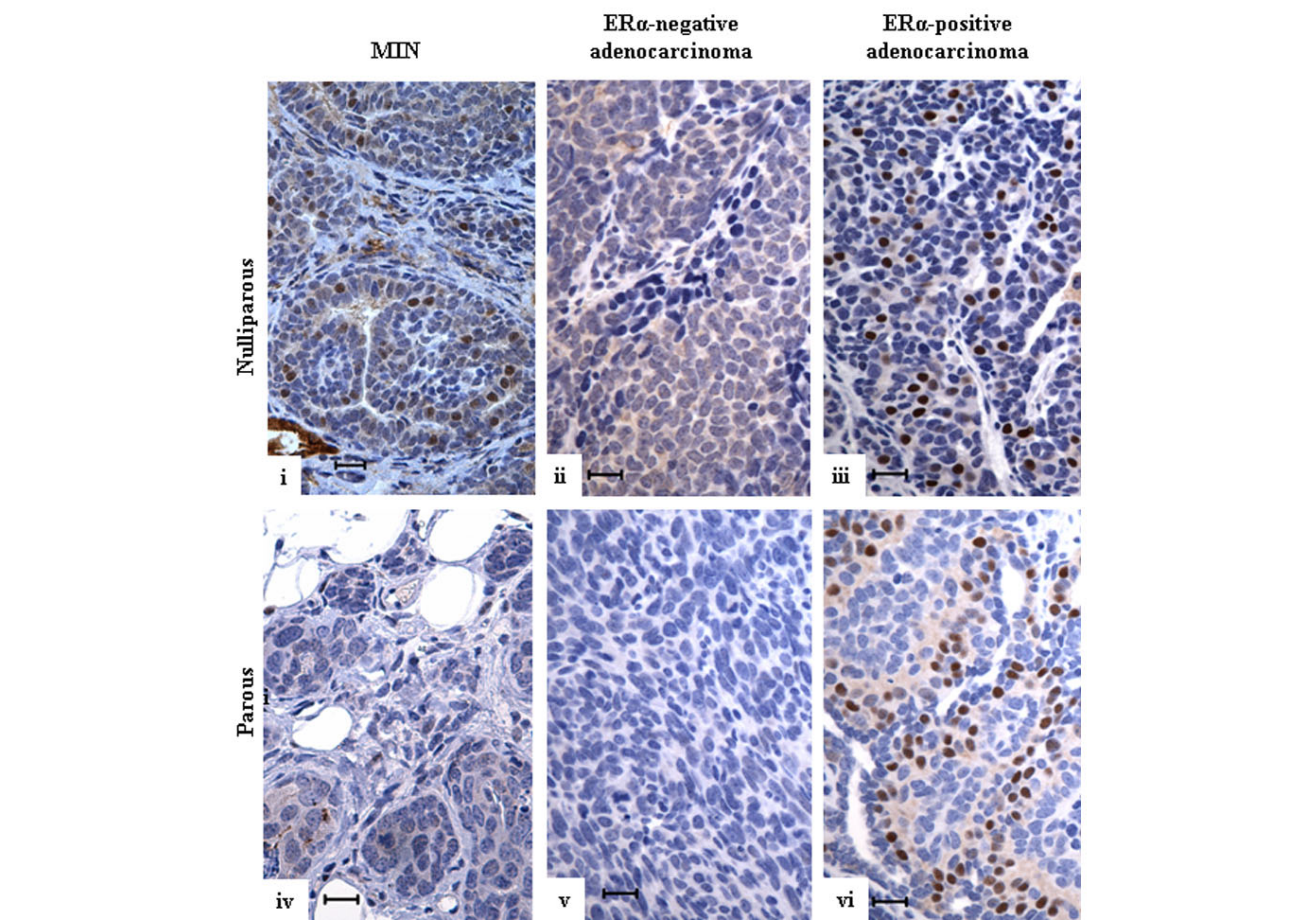
Estrogen receptor-alpha expression in spontaneous mammary tumors in *Trp53*^+/- ^mice. Spontaneous mammary tumors from nulliparous **(i-iii) **and parous **(iv-vi) **BALB/c-*Trp53*^+/- ^mice were stained with anti-ERα antibodies. Representative sections are shown for mammary intra-epithelial neoplasias (MIN) and adenocarcinomas that differ in ERα status. The tissues from nulliparous mice include **(i) **ERα^+ ^MIN, **(ii) **ERα^- ^adenocarcinoma, and **(iii) **ERα^+ ^adenocarcinoma. The tissues from parous mice include **(iv) **ERα^- ^MIN, **(v) **ERα^- ^adenocarcinoma, and **(vi) **ERα^+ ^adenocarcinoma. Scale bar = 20 μm. ERα, estrogen receptor-alpha.

The histopathology and ERα status of the tumors were also examined to determine whether parity altered the subtypes of tumors in the BALB/c-*Trp53*^+/- ^mice. Adenocarcinomas represented the dominant histopathological class of tumors in both nulliparous and parous mice (Table 1) (86.2% and 87.5%, respectively). Although mammary intra-epithelial neoplasias (MINs) are considered precursors of adenocarcinomas, these were a small proportion of tissues because only palpable lesions were collected. Carcinosarcomas and adenosquamous tumors were infrequent. Expression of ERα was detected in MIN and adenocarcinomas (Figure 7). Among adenocarcinomas, the proportion positive for ERα was increased from 12% among the nulliparous group to 33% among the parous group (Table 1) (*P *< 0.001). The increase in ERα^+ ^tumors may reflect a difference in the cellular origins of the tumors between nulliparous and parous mice. However, this may be confounded with the fact that the mammary tumors in older mice are often collected at an earlier stage of tumor progression because of comorbidities (for example, lymphomas). The mammary tumors in BALB/c-*Trp53*^+/- ^mice appear to progress from ERα^+ ^lesions to ERα^- ^tumors (D.J. Jerry, unpublished data). Thus, the difference in ERα expression may reflect an earlier stage of progression among mammary tumors from parous mice due to the longer latencies.

**Table 1:** Characteristics of spontaneous mammary tumors in BALB/c-*Trp53*^+/- ^mice

Reproductive history	Histopathology	Number	Frequency	ERα-positive
Nulliparous	Mammary intra-epithelial neoplasia	1	3.4%	1
	Adenocarcinoma	25	86.2%	3
	Carcinosarcoma	1	3.4%	0
	Adenosquamous	2	6.8%	0

Parous	Mammary intra-epithelial neoplasia	2	8.3%	0
	Adenocarcinoma	21	87.5%	7
	Carcinosarcoma	0	0	0
	Adenosquamous	1	4.2%	0

ERα, estrogen receptor-alpha.

### The protective effect of parity and E+P treatment is not retained in epithelial transplants

Parity offers lifetime resistance to mammary tumorigenesis despite the fact that the parous epithelium will go through repeated cycles of proliferation and regression at each estrus cycle and with each pregnancy. Therefore, the protective effect may be programmed into mammary epithelial cells, and specifically mammary epithelial progenitor cells. If this is the case, mammary epithelium from a hormone-stimulated donor transplanted into the cleared fat pad of a nulliparous host should retain protective features. Therefore, we tested whether the apoptotic response to radiation was retained by the epithelial outgrowths that originate from E+P-treated or parous tissue (Figure 8a). There was no difference in radiation-induced apoptosis between *Trp53*^+/- ^epithelial outgrowths from nulliparous, E+P-treated, or parous donors. Apoptosis in *Trp53*^+/- ^epithelial outgrowths (Figure 8b) did not differ from the *Trp53*^+/- ^nulliparous epithelium from glands of ovary-intact mice (Figure 6a). Although E+P treatment enhanced radiation-induced apoptosis in donor tissues (Figure 6a), apoptotic responses to radiation were significantly lower among outgrowths of *Trp53*^+/- ^mammary epithelium regardless of the treatment of the donor tissue (Figure 8b). Thus, the outgrowths failed to retain the sensitivity to radiation that was present in the donor tissue.

To determine whether transplanted epithelium from E+P-treated donors may retain the protective effects of the hormone stimulation after transplantation into the cleared fat pad of nulliparous hosts, we compared tumor incidence and latency between glands bearing transplants of *Trp53*^+/- ^mammary epithelium from nulliparous versus E+P-treated donor mice. All tumors from the transplanted epithelium, whether E+P-treated or nulliparous, developed adenocarcinomas with similar histopathological features. There was no significant difference in tumor incidence or latency between the epithelium from the E+P-treated donor or the nulliparous donor (Figure 9). Because mammary tumorigenesis is not reduced in the E+P-treated epithelium compared with the nulliparous epithelium and because the radiation-induced apoptotic response is not retained by the hormone-stimulated outgrowths, the parity-induced changes are not a stable phenotype retained by the epithelial progenitor cells that repopulate the gland.

## Discussion

The significant role of p53 in tumor suppression in the mammary epithelium was demonstrated by the frequent occurrence of spontaneous tumors in transplants of BALB/c-*Trp53*^-/- ^mammary epithelium [22]. As p53 protein promotes cell cycle arrest and apoptosis, both can play significant roles in tumor suppression. However, only the proapoptotic function is required to prevent lymphomas whereas the cell cycle checkpoint activity is dispensable [33]. Therefore, enhancement of apoptosis in the mammary epithelium appears to be a critical activity for suppression of tumors. Both radiation-induced and spontaneous apoptosis were enhanced in parous and E+P-treated mammary epithelium (Figures 3c and 3d). As radiation-induced apoptosis was 2.5-fold higher in *Trp53*^+/+ ^mammary tissues than the *Trp53*^-/- ^after hormonal stimulation (Figure 6a), the majority of apoptosis is p53-dependent. Though lower in magnitude, apoptosis in the mammary epithelium of *Trp53*^-/- ^mice was also increased by E+P treatment (Figure 6a). Therefore, in addition to enhancing p53 function, hormonal exposures appear to alter the promotional environment of the host to enhance p53-independent apoptosis pathways [34], both of which may contribute to tumor suppression.

Inducible expression of p53 in mouse models has shown that even short-term expression of p53 can substantially delay the appearance of lymphomas and liver carcinomas [35,36]. Therefore, the duration of the enhancement of p53 was of interest. Treatment with E+P for 14 days stimulated changes in the mammary epithelium such that p53-dependent apoptosis in the mammary epithelium persisted even after ovariectomy, indicating that continued exposure to ovarian hormones is not necessary to maintain p53 activity. These results imply that neither the nulliparous nor parous glands are necessarily more vulnerable to genotoxic stress at one phase of the estrus cycle or another. Rather, there is an enhancement of apoptosis that is sustained long after pregnancy and even after cessation of ovarian function.

Breast cancer is the most common tumor among females with Li-Fraumeni syndrome, which is most often associated with heterozygous mutations in the gene encoding p53 [28], underscoring the prominent role of *TP53 *in determining breast cancer susceptibility. However, it is not clear whether the risk of breast cancer among women with Li-Fraumeni syndrome is due to reduced activity of p53 or the increased risk of losing the wild-type allele of *TP53*. As levels of radiation-induced apoptosis in *Trp53*^+/- ^mouse mammary epithelium were intermediate to the responses in *Trp53*^+/+ ^and *Trp53*^-/- ^tissues (Figure 6a), *Trp53*^+/- ^mammary tissues are haploinsufficient with respect to apoptosis. This intermediate apoptotic response phenotype is consistent with apoptosis observed in *Trp53*^+/- ^mice during involution of the prostate following castration [37]. Stress responses were also attenuated in HCT116 cells that were heterozygous for *TP53 *compared with the wild-type cells [38]. Thus, the activity of p53 may fall below a threshold in *Trp53*^+/- ^mammary epithelium, as proposed by Santarosa and Ashworth [39], to a point that is no longer sufficient to engage appropriate apoptotic responses to genotoxic stress. The appearance of spontaneous mammary tumors was delayed among parous BALB/c-*Trp53*^+/- ^mice (Figure 6b). Therefore, the activity of p53 is reduced to critical levels in the mammary epithelium of BALB/c-*Trp53*^+/- ^mice, but hormonal stimulation increases p53-dependent apoptosis and renders the tissue more resistant to tumorigenesis. As parity did not prevent tumors in BALB/c-*Trp53*^-/- ^mammary epithelial transplants [22], the decrease in mammary tumors among parous BALB/c-*Trp53*^+/- ^(Figure 6b) appears to be largely p53-dependent.

The mechanism by which E+P sensitizes the p53 pathway is initiated by their respective receptors [27]. In women, the protective effects of parity are limited to the ERα^+^/progesterone receptor-positive tumors [40]. Thus, we investigated whether parity diminished the proportion of ERα^+ ^tumors in BALB/c-*Trp53*^+/- ^mice. In contrast to expectations, the proportion of ERα^+ ^tumors was increased among parous mice (Table 1). ERα^+ ^mammary tumors were also observed in C57BL/6J mice rendered p53-deficient by inducible deletion by WAP/Cre [41]. However, these results should be interpreted with caution because ERα status is confounded with age of mice. Since mammary tumors in BALB/c-*Trp53*^+/- ^mice progress from ERα^+ ^lesions to ERα^- ^tumors (K.A. Dunphy, A.C. Blackburn, H. Yan, L.R. O'Connell, D.J. Jerry, unpublished data), the ERα expression observed among mammary tumors in parous mice may reflect an earlier stage of progression due to the longer latencies. Additional markers to distinguish basal-like and luminal properties will be needed in order to ascertain whether the cellular origins differ for the mammary tumors in nulliparous and parous BALB/c-*Trp53*^+/- ^mice.

Decreased expression of ERα and increased expression of ERβ are persistent changes observed in mammary epithelium of parous rodents [8]. ERα and ERβ exert antagonistic effects on p53 activity [42,43]. This could explain the increased p53-dependent apopotosis in hormone-stimulated mammary tissues. Alternatively, the persistent phenotypic changes in ERs and responsiveness of p53 may reflect alterations in the fates of the progenitor cell pool. Indeed, parity-induced mammary epithelial cells have been identified and serve as progenitors of the lobulo-alveolar structures during subsequent pregnancies [44,45]. However, outgrowths of *Trp53*^+/- ^mammary epithelium from E+P-treated and parous mice failed to retain the elevated apoptotic response to radiation in nulliparous hosts (Figure 9). The characteristics of the outgrowths were not different from nulliparous transplants or nulliparous glands (Figure 6a). Likewise, the outgrowths of E+P-treated *Trp53*^+/- ^mammary epithelium developed tumors with frequency and latency similar to those of the *Trp53*^+/- ^transplants from nulliparous mice (Figure 9).

These observations do not preclude the possibility that the fates or number of stem cells may be decreased within the mammary glands of hormone-stimulated mice compared with nulliparous mice. It remains possible that parity decreases the pool of progenitor cells [46] but does not limit the capacity of the stem cells to repopulate upon transplantation. It is equally possible that hormonal stimulation imparts a stable change in the stromal cells and extracellular matrix which modulate p53 function. The extracellular matrix from involuting glands can promote tumors [17,18] but also is essential for differentiation [47,48], and parous stroma decreased the incidence of tumors [49]. Many extracellular matrix proteins change dramatically across developmental phases and can alter integrin-mediated signaling in the mammary gland [19,50,51]. Loss of integrin signaling results in apoptosis in the mammary epithelium [52]. Therefore, hormonal stimulation may result in stable changes within the stroma which limit either tumorigenic transformation of progenitor cells or their progression to invasive tumors.

## Conclusion

These results demonstrate that E+P and parity enhance p53-dependent responses to radiation to a similar extent. Increased p53 surveillance and clearance of compromised cells by apoptosis is likely a critical activity that reduces tumors in parous individuals. The heightened sensitivity of p53 is sustained even after withdrawal of ovarian hormones. While the parity-influenced apoptotic mechanism is intrinsic to the mammary gland itself, it is not permanently programmed into the epithelial progenitor cells. Thus, the heightened surveillance of parous mammary epithelium was lost when transplanted into cleared mammary fat pads of nulliparous mice and the transplanted epithelium acquired the tumor incidence of the host. This suggests a mechanism in which the hormones act on the stroma as well as the epithelium to communicate reciprocally to enhance p53-dependent apoptosis.

## Abbreviations

AMV = age-matched virgin; E+P = estradiol and progesterone; ER = estrogen receptor; DMEM = Dulbecco's modified Eagle's medium; MIN = mammary intra-epithelial neoplasias; *Trp53 *= transformation-related protein 53 (gene in mouse encoding the p53 tumor suppressor protein); TUNEL = terminal uridine deoxynucleotidyl transferase dUTP nick-end labeling.

## Competing interests

The authors declare that they have no competing interests.

## Authors' contributions

KAD was responsible for designing and carrying out the bulk of the experiments as well as drafting the manuscript. ACB and HY provided the data on tumor phenotypes in *Trp53*^+/- ^mice. LRO participated in the transplant studies. DJJ was responsible for the design and coordination of the experiments as well as preparation of the manuscript. All authors read and approved the final manuscript.
